# Framework for the Development and Delivery of Digital Peer Support Programs: Qualitative Study on in-Person and Digital Delivery for People With Cardiovascular Disease

**DOI:** 10.2196/72743

**Published:** 2025-10-16

**Authors:** Joseph Weddell, Emily Li, Wendan Shi, Robyn Gallagher, Stephanie Ruth Partridge, Karice Hyun, Vanessa Poulsen, Christian Verdicchio, Thomas Buckley, Julie Redfern

**Affiliations:** 1 Sydney Nursing School, Faculty of Medicine and Health, University of Sydney, Sydney, Australia; 2 Charles Perkins Centre, Faculty of Medicine and Health, University of Sydney, Level 2 2W11, Building D17, Sydney, 2006, Australia, 61 02 8627 1616; 3 Sydney School of Health Sciences, Faculty of Medicine and Health, University of Sydney, Sydney, Australia; 4 Cardiology Department, Concord Hospital, Anzac Research Institute, Concord, Australia; 5 National Heart Foundation of Australia, Adelaide, Australia; 6 Heart Support Australia, Canberra, Australia; 7 Centre for Heart Rhythm Disorders, University of Adelaide, Adelaide, Australia; 8 Institute for Evidence-Based Healthcare, Faculty of Health Sciences and Medicine, Bond University, Gold Coast, Australia

**Keywords:** peer support, peer group, cardiovascular disease, focus groups, coronary heart disease, digital health, mobile apps, cardiac rehabilitation

## Abstract

**Background:**

Peer support (ie, sharing experiences and providing support with others with the same condition) improves health outcomes among people with cardiovascular disease (CVD), including self-management behaviors and self-efficacy. However, current peer support interventions are diverse, and evidence is lacking on the perceptions of benefits and the elements considered priorities by peer support attenders, especially regarding digital interventions.

**Objective:**

The study aimed to (1) describe perceived benefits and recommendations for CVD peer support programs from people attending in-person peer support, (2) identify priorities for digital peer support from consumers and clinicians testing a peer support app prototype, and (3) develop a framework to inform future peer support intervention development.

**Methods:**

A qualitative study design was used across 2 components to address the objectives. In Component 1, semistructured focus groups were conducted with attenders of established in-person CVD peer support groups to explore the perceived benefits of peer support and recommendations for future programs. In Component 2, semistructured workshops with consumers with CVD and semistructured interviews with CVD clinicians/researchers were conducted to obtain feedback and recommendations for digital peer support using an exploratory digital CVD peer support application prototype. Data were digitally recorded, transcribed verbatim, and analyzed thematically. Findings from both components were iteratively synthesized to inform the development of a digital peer support framework.

**Results:**

In Component 1, a total of 22 participants (age range 29‐84 years, 45% male) took part in focus groups. The overarching theme was that peer support provides benefits through the sharing of experiences. Five themes were refined and defined: (1) peer support provides a way of coping; (2) peers learn from each other; (3) peers understand what each other is going through; (4) the peer community uplifts mood and builds confidence; and (5) awareness, flexibility, and resources are important for engagement. In Component 2, five participants (age range 55‐74 years, 60% male) attended 2 workshops, and 8 clinicians-researchers (age range 30‐65 years, 10% male) were interviewed. Three themes were refined and defined: (1) autonomy is essential to promote engagement; (2) safeguarding is important to both users and clinicians; and (3) interfaces that are simple, easy to use, and visually attractive enable use. Priorities identified from both components included greater peer support awareness and uptake, flexibility with timing and family participation, health care professional involvement, provision of resources, autonomous features enabling choice, checklists and clinician moderation for safeguarding, and simple-to-use interfaces.

**Conclusions:**

Participants in peer support programs derive benefit from sharing their experience of living with CVD, which enables coping, learning, feeling understood, and a sense of community. Priorities were synthesized to create a framework for digital peer support development, with recommendations to focus on 6 key areas: uptake, flexibility, resources, autonomy, safeguarding, and interface.

## Introduction

Cardiovascular disease (CVD) is the leading cause of death worldwide, with approximately 20.5 million people reportedly dying every year [1]. In acute coronary syndrome, a manifestation of coronary heart disease (CHD), the readmission rate is approximately 22%, with 69% of these readmissions occurring more than once within 1 year [1,2]. Many of these readmissions are preventable with secondary prevention strategies, including lifestyle modification, risk factor reduction, medication adherence, and regular exercise. These strategies also contribute to improved quality of life and decreased morbidity and mortality [3]. International guidelines acknowledge that CVD secondary prevention is most effectively delivered through cardiac rehabilitation, traditionally consisting of group-based exercise, education, and counseling sessions delivered face-to-face (in-person), which also provide opportunities to address psychosocial issues that are common after a heart attack [4]. In-person cardiac rehabilitation may be effective because participants can interact with and benefit from others with cardiac conditions [5,6]. However, low attendance and completion is a worldwide persistent issue [7]. Since the COVID-19 pandemic, many providers have adopted virtual, home-based cardiac rehabilitation models [8], which are nonetheless still subject to limitations in uptake [9] and may lack the peer interaction benefits of in-person programs. Peer support provides an alternative or complementary support to in-person or home-based cardiac rehabilitation, offering opportunities to interact with others [10].

Social support is acknowledged as an important component of recovery in chronic conditions [11], especially after acute coronary syndrome [12]. However, social support from overprotective family and friends can be counterproductive [13], and support from health care professionals is often constrained, with clinicians sometimes lacking specific lived experience. Given these limitations, peer support, which encompasses lived experience, strategy, and encouragement sharing between people who have the same condition [10]. Peer support is distinct from other forms of support in that experiential knowledge shared by people with lived experience of the condition often has higher credibility to the receiver [14] and typically results in optimism and confidence to manage a condition similarly. Moreover, people who share similarities (eg, diagnosis and treatment, including surgical intervention) often feel more comfortable interacting with one another [15]. Benefits are bidirectional, with those providing support also gaining advantages [14]. Peer support strategies are diverse but have overall been shown to improve quality of life, self-management behaviors, and self-efficacy in rheumatic diseases [16], traumatic injury [17], type 2 diabetes [18], and cancer [19], largely through in-person, group-based formats. More recently, digital peer support interventions have demonstrated usefulness in chronic conditions, including mental health [20], adolescent cancer [21], and type 2 diabetes [22].

Among people with a cardiac condition, peer support has been reported to provide several benefits, including increased exercise participation, smoking cessation, wider peer networking, and higher levels of nonfamily social support [23]. A recent systematic review and meta-analysis categorizing CHD peer support interventions by format demonstrated that peer support interventions (including in-person, via telephone, or messaging) are effective in increasing self-efficacy and self-management behaviors and reducing readmissions among people with CHD [24]. However, no randomized controlled trials evaluating digital peer support interventions for people with CHD were identified [24]. Furthermore, components of digital peer support interventions, such as content development, engagement strategies, the role of moderation, and underlying theory, require further exploration [25]. Studies evaluating the efficacy of peer support interventions are diverse and heterogeneous [26], and there is limited research investigating the feasibility and acceptability of peer support for people with CVD, including the components that work best for peers and the perceived benefits of participation in such interventions [24]. Therefore, this study aimed to understand the perceived important benefits and recommendations of peer support from people attending in-person peer support programs (Component 1) and from consumers and clinicians testing a digital peer support app prototype (Component 2). A further aim was to iteratively synthesize recommendations identified in Components 1 and 2 to develop a framework and recommendations for future peer support program development.

## Method

### Study Design

This study used a qualitative design to collect data for the 2 components. For Component 1, two semistructured focus groups were conducted between October and November 2022 to explore the benefits of peer support and preferences for future strategies within 2 established heart disease peer support groups, based in New South Wales and the Australian Capital Territory, Australia. For Component 2, two semistructured consumer workshops and 8 semistructured interviews were undertaken between August and September 2020, in which a CVD digital peer support app prototype was tested by consumers (workshops) and clinicians/researchers with expertise in CVD (interviews). The workshops and interviews were used to generate feedback for future digital peer support intervention preferences.

### Eligibility Criteria

Participants for Component 1 were eligible if they (1) had a self-reported diagnosis of heart disease, or were the caregiver of someone with heart disease, and (2) attended a peer support group for heart disease, and (3) were older than 18 years of age. Participants for Component 2 were eligible if they were people (excluding caregivers) who met criteria 1 and 3, or were clinicians and researchers with expertise working with patients with CVD. In Component 1, caregivers were specifically included, along with consumers with heart disease, as these people are often regular attenders of peer support groups. However, caregivers were excluded from Component 2 because this phase was exploratory, testing a digital peer support application prototype, with the investigators specifically aiming to explore the lived experience perspectives of people with heart disease regarding application use. Therefore, at that stage, a small pilot sample without caregivers was deemed to be the most appropriate method for facilitating a small consumer workshop. Individuals lacking proficiency in the English language were excluded.

### Sample and Setting

For Component 1, participants were recruited from 2 established in-person peer support groups managed by Heart Support Australia. This organization manages 26 consumer-led peer support programs across Australia, which typically meet monthly or more, each facilitated by a trained peer support group leader who has lived experience of CVD. The focus of these peer support programs is to encourage self-management and provide ongoing support for people with heart disease [27]. The 2 groups approached for this study were based in separate geographical locations (New South Wales and the Australian Capital Territory), with one in a metropolitan location and one in a large regional town. Participants were recruited using convenience sampling through the peer support leaders of each group, respectively, who approached regular attenders and asked if they would be interested in participating in focus groups on a specified forthcoming date. On that date, coinciding with the regular peer support group meeting, the research team attended and recruited participants for the study. Data collection commenced immediately.

For Component 2, the 2 consumer workshops were held at the University of Sydney, New South Wales, and interviews with CVD experts were conducted via the Zoom online communication platform (version 5.7; Eric Yuan). Participants for the consumer workshops were purposively recruited from a database of participants of a previous CVD study who had expressed interest in future research involvement. Clinicians and researchers were recruited purposively through networks of professionals working in CVD, with a range of ages, disciplines, and clinical experience sought. Written consent was obtained for all participants across both components before data collection commenced.

### Procedure

Potential participants who were eligible and expressed interest were invited to take part in focus groups (Component 1), consumer workshops, or interviews (Component 2), respectively. Focus groups and workshops lasted approximately 60 to 80 minutes, and semistructured interviews lasted approximately 30 to 45 minutes. Two experienced facilitators were present in each focus group and workshop, with one facilitating the discussion and the other taking field notes. Semistructured interviews were conducted by an experienced researcher. Facilitators had completed PhDs and had experience undertaking qualitative research, and the facilitators leading discussions in each group were also clinicians with expertise in CVD. The facilitators had no existing clinical relationships with any of the participants from the study and no potential to provide future treatment or support to the participants. After making introductions, facilitators discussed confidentiality and recording with participants, ensured all voices were heard, and then followed a semistructured discussion guide.

Component 1, the discussion guide, included how participants found out about peer support, what motivated attendance, what participants liked or gained from peer support, operational questions (ie, frequency and duration of each meeting), and recommendations for peer support programs (including preferences for digital delivery). The Component 2 discussion guide included participant perceptions about the usefulness of a peer support app for self-managing CVD, enabling social support, and sharing experiences, when it should be applied (timing), the potential role of health care professionals, and general impressions of the prototype, including navigation, usability, appeal, and content. All focus groups and interviews were digitally recorded and transcribed verbatim. Data were deidentified by the investigators after transcription and before analyzing the data, with participant numbers used in reporting to ensure confidentiality.

### Analysis

Data were analyzed by thematic analysis as outlined by Braun and Clarke [28]. As each component had differing aims, purposes, and data collection approaches, differing theoretical approaches were used to determine data saturation, as proposed by Saunders et al [29]. For Component 1, an inductive thematic saturation (the Second Model of Data Saturation of Saunders et al [29]) was used, whereby data saturation was determined by the achievement of no further emerging codes or themes. Due to the exploratory nature of Component 2 and the testing of a prototype CVD peer support app in small consumer workshops and one-on-one interviews with clinicians, we followed the Fourth Model of Data Saturation of Saunders et al [29]. In this model, saturation is determined by redundancy in the data, rather than a lack of emerging themes or specifically in relation to an underlying theory, and is therefore distinct from formal data analysis [29]. The investigators engaged in robust discussion around reflexivity before data analysis, including their gender, clinical backgrounds, qualifications, and the potential impact of their experiences. The authors who conducted the interviews were female and held doctoral research degrees [RG and EL]. For each component, 3 investigators [JW, EL, and WS] independently reviewed all transcripts and recordings before collating data into initial codes. Codes were then developed into themes and subthemes individually before being compared collaboratively between these investigators. Then, using an iterative process, themes and subthemes were reflected on, reworked, and refined, with a fourth investigator [JR] consulted for consensus. Themes and subthemes are presented with verbatim quotations, alongside participant pseudonym initials, age, and sex. Exemplar quotations demonstrating the process of coding are provided in Multimedia Appendix 1. Preferences identified in Components 1 and 2 were synthesized to inform a series of recommendations for future digital peer support programs and research.

### Ethical Considerations

Ethics approval was granted by the University of Sydney Human Research Ethics Committee (2020/515). An ethics amendment has since been submitted and approved to account for additional investigators who were added to the study to assist with secondary analysis. Participants were provided with a participant information sheet, fully explaining the purpose of the study. Written informed consent was collected from all participants who agreed to take part. Following collection, all data were safely and securely stored in protected institutional servers with multifactor identification security and restricted access. Data were deidentified by the primary author before review by the wider coauthorship team. Participants for Component 1 were offered an Aus $25 gift card (approximately €15 and US $15) to help cover related expenses. Participants in Component 2 were reimbursed for parking fees and provided lunch and refreshments of approximately the same value. The study conforms with the Declaration of Helsinki and is reported according to the items of the COREQ (Consolidated Criteria for Reporting Qualitative Research) checklist ([30]; Checklist 1).

## Results

### In-Person Peer Support

For Component 1, 22 participants took part in the 2 focus groups. The majority were in the age group of 75‐84 years (45%), male (59%), married or partnered (73%), and retired (82%; Table 1). An overarching theme identified was that peer support provides benefits through shared experiences. Five main themes were refined and defined: (1) peer support provides a way of coping; (2) peers learn from each other; (3) peers understand what each other is going through; (4) the peer community uplifts mood and builds confidence; and (5) awareness, flexibility, and resources are important for engagement (Textbox 1).

**Table 1.** Characteristics of participants in Component 1 (n=22; in-person peer support).

Participant	Value, n (%)
Age range (years)	
<45	1 (5)
45‐54	2 (9)
55‐64	3 (14)
65‐74	6 (27)
75‐84	10 (45)
Sex	
Male	13 (59)
Education	
Secondary education only	6 (27)
Diploma	5 (23)
Undergraduate	5 (23)
Postgraduate	6 (27)
Marital status	
Married or partnered	16 (73)
Single or separated	5 (23)
Widowed	1 (5)
Employment	
Retired	18 (82)
Unemployed	3 (14)
Employed	1 (5)

Textbox 1. **Themes and subthemes of Component 1 (in-person peer support).**

**Theme 1–Peer support provides a way of coping**

- Helps to deal with psychological impacts
  *“He said, I felt really awful, I kept telling my wife I was depressed. I said, well, being depressed afterwards or feeling low is absolutely normal. And he said, Oh, really? No one had actually told him before, and he didn’t have the benefit of a group like this.*” [Participant 2, male]

- Talking about experiences provides “cathartic relief”
  *“….looked at from a peer support, it means you’ve got a group of people who can get together, basically share their experience, and just let it out. And I think that forms a really unique sort of bond.*” [Participant 15, male]

- Hearing from peers helps normalize individuals’ own experiences
  *“You feel a bit guilty that you weren’t doing the right thing. When you look around [the peer support group], you look at people and think, well, they were the same as me. It makes you feel better about yourself, obviously. And you’re not so hard on yourself about things.*” [Participant 7, male]

**Theme 2–Peers learn from each other**

- You find out from peers what “nobody bothered telling me”
  *“….they took about two and a half feet of vein out of my left leg. And they didn’t say anything much about that at all. Some weeks afterwards, I was having an issue, not with the heart, but where they took the graft out, it was numb and tingling.*” [Participant 2, male]
- Peer support helps make sense of hospital admission
  *“I had my first inkling at about half past eight in the morning, I was in surgery by half past 12, and back home the following day. My biggest problem was saying, What the hell happened? There was no time for people to impart information, or for me to take that information in and synthesize it, and come up with the logical reasoning.*” [Participant 9, male]
- Peer support fills shortcomings in patient education
  *“There is a massive hole in the medical profession for that education, firsthand knowledge, whatever. This fills it. But that medical side, they can’t….I don’t think they’ve got the resources. I don’t think they’ve got the time.*” [Participant 17, male]

**Theme 3–Peers understand what each other is going through**

- We do not have to worry about family unnecessarily
  *‘There are obviously things that it’s better if you [unload] to somebody totally outside of the family. This is where I think it’s important to have the contact. You can just pick up the phone and go, I need to talk to someone, and you’re not bothering…. you’re not worrying your family unnecessarily. You can talk to somebody who’s been through the same thing, but your family isn’t getting all concerned. I think that’s important.*” [Participant 15, male]
- Experience and advice are valued because they come from a peer
  *“It’s just nice to meet up people who’ve been through [the same thing], you know what I mean? When I had a cancer, I got some [peer] support from the Cancer Council for the grief. So when they said there was a heart one, I thought, I wonder...I’ll try it out.*” [Participant 20, female]
- Peer support is a safe space for asking questions
  *“We all come to an understanding fairly quickly: there are no stupid questions. If you’ve got a question, it’s obviously worrying you, so it can’t be stupid. I think we all understand that. So, the questions get asked and some can be quite personal, and that doesn’t matter.*” [Participant 9, male]

**Theme 4–The peer community uplifts mood and builds confidence**

- “People pick you up”
  *‘That’s how I feel about [the peer support] group. Sometimes you don’t say anything....you don’t need to ask questions or say how your feelings are, because by the end of the group session, you’ve gotten some information or you feel a little bit light-hearted, and lifted by being around….*[Participant: *or somebody asks you….*] *That’s right! ‘Are you okay?’’’* [Participant 6, female]
- Experiences provide confidence, hope, and vicarious self-efficacy
  *‘The good thing about it is there’s people more advanced….they’ve had heart attacks long time ago, so we can learn off them just what’s in store for us. And seeing somebody, excuse me saying so, old. You’re not worried about your life so much. It’s not a death sentence.*” [Participant 7, male]
- The group helps support each other
  *“If someone’s not here, we usually know. Someone usually knows where they are. [Interviewer: someone’s checked in on them?] Yeah. So that’s sort of reassuring. That kind of family aspect….*” [Participant 5, male]

**Theme 5–Awareness, flexibility, and resources are important for engagement**

- Greater peer support awareness is required for uptake
  *“I actually Googled to find this group….I just had a defibrillator but I had a frozen shoulder after my six weeks of not being able to lift my arms and all that. I found it really difficult. I kept going back to my doctor and she said she’d ring the rehab, and they said, no, we don’t take people who just have defibrillators. I was like, well, what do I do?*” [Participant 14, female]
- Flexibility with timing and family involvement is desirable to maximize reach
  *“[some people with CVD] still have to work. They still have to mind their children at home or their grandchildren. They haven’t got that one day a month that they can go okay, I can definitely do it on the Tuesday. I could definitely do it on Wednesday.*” [Participant 6, female]
- Health professional involvement provides a talking point
  *“We’ve had doctors come and talk about medication or you know, have set up life plans and all sorts of different things that, you know, we all call us are on medication. So having that information given to us helps. Cause you know, you, are in hospital, they give you a bag of medication and say, Here you go! So you can kill yourself on this!*” [Participant 17, male]
- Supplementary materials enhance discussions and experience sharing
  *“When we’ve got the information on pamphlet and we sit around talking about it, it gives you more support for each other. Handouts are always good. You can take them home and read it later again or refer to what someone in your group had said, I’ll read about that.*” [Participant 6, female]

#### Theme 1: Peer Support Provides a Way of Coping

Through sharing experiences in a variety of ways, including using humor, discussing both similar and different stories, and talking about their concerns, participants were able to find a variety of coping strategies they could use to help with challenges they faced in their CVD recovery. For some, this enabled acceptance of these challenges, helped by having “people around who you can talk to.” Some participants reported that experience sharing enabled them to be cognizant of and address a previous reluctance to ask for support. This was particularly relevant when participants talked of overcoming the isolating nature of psychological challenges after CVD diagnosis. For others, peer support participation provided an emotional outlet, in which participants acknowledged that the process of sharing experiences “is a cathartic one.” Experience sharing also resulted in people realizing “they’re not alone,” with many participants finding common ground in the issues they had been through in their recovery through peer support attendance.

#### Theme 2: Peers Learn From Each Other

Peer support enabled a forum for open discussion in which participants gleaned advice, guidance, and information from each other’s experiences, and participants were able to “learn new things every day.” The peer support environment was useful for understanding specific conditions, challenges, and symptoms because participants could simply ask for advice or take guidance from another peer’s shared experience. This was particularly beneficial for those participants who underwent coronary angioplasty and had short hospital admissions versus those who had, for example, undergone coronary artery bypass grafts (typically requiring 1 week in hospital), who “at least had time to get my head around this.” For some, the short admission highlighted a lack of information provided, and for others, insufficient time to process the information received during admission. Participants, therefore, identified peer support as an important resource for learning, particularly regarding the hurdles of posthospital recovery that health care professionals did not inform them about. This highlighted inconsistencies in the information priorities of people with CVD compared with information that health care professionals consider to be pertinent. For participants with specific CVD diagnoses, for example, implanted cardiac defibrillators, peers often sought the experiences of others with the same specific condition, especially related to how to seek advice.

#### Theme 3: Peers Understand What Each Other Is Going Through

The peer support setting fostered an environment where peers felt a shared sense of understanding “with like-minded people,” where participants were “understood and appreciated for that insight.” This environment, where everyone had gone through a similar process, promoted the sharing and valuing of opinions and experiences between peers. Participants acknowledged that this shared understanding encouraged a forum to raise questions and discuss issues without having to worry about family members or friends, who may overreact to or not comprehend symptoms or feelings. Peer support also avoided feelings of societal judgment arising from discussing challenges with people without CVD, who participants reflected “don’t understand.” As a result, peers considered their group to be a safe space for asking questions and sharing insights, especially things deemed inappropriate for health care professionals and family members. This included taboo or judgmental issues (eg, weight, diet, and alcohol), and “stupid questions” perceived as too trivial or simple to ask professionals.

#### Theme 4: The Peer Support Community Uplifts Mood and Builds Confidence

Participants described peer support as a community environment where rapport and camaraderie were fostered. This sense of community had a direct influence on their mood and confidence in every peer support session. Participants reported that when individuals attend in a bad or worried mood, they are often positively swayed by the group dynamic, and that “you can come in here, could be any reason you’re having a bad day, and then people pick you up.” Being able to interact with peers who are further ahead or “more advanced” in their recovery journey allowed participants to develop hope for the future and the confidence to manage their conditions, vicariously building self-efficacy. The peer support environment encouraged a sense of camaraderie, where peers developed friendships, swapped phone numbers, “checked up,” and supported members outside of the peer group sessions.

#### Theme 5: Awareness, Flexibility, and Resources Are Important for Engagement

Participants recognized a need for greater peer support awareness, both from the general public and health care professionals. Awareness of peer support was acknowledged to often be dependent on advocacy from peer volunteers who visited CVD inpatients during hospital admission. When this did not occur, participants reflected that hospital staff often overloaded patients with information and resources, but not always including peer support group information. Participants acknowledged that awareness and uptake are important considerations because a critical “mass of people” is required for engagement, and that “it won’t work if you haven’t got many people turn up to peer group meetings.” To encourage peer support attendance, participants reported a need for flexibility around when sessions are offered and for whom, including throughout all stages of recovery and for family members and partners too. Participants highlighted that the involvement of health care professionals to deliver talks and distribute additional resources enhanced peer support sessions and improved engagement, as this provided talking points and allowed participants to connect with the content of the materials and with each other via sharing experiences.

### Digital Peer Support

In Component 2, there were 5 participants in 2 consumer workshops. The majority were in the age group of 65‐74 years (60%), male (60%), married or partnered (80%), and retired (80%; Multimedia Appendix 2). A total of 8 CVD clinicians and researchers were interviewed, including 2 registered nurses, a cardiologist, a dietitian, an exercise physiologist, a physiotherapist, an epidemiologist, and a public health researcher. Most of these professionals were in the age group of 55‐65 years (38%) and female (90%), with a mean work experience of 11.6 (SD 5.9) years (Multimedia Appendix 2). While recommendations for individual elements of digital peer support interventions varied between consumers and professionals, 3 overall themes were refined and defined: (1) autonomy is essential to promote app engagement; (2) safeguarding is important to both users and clinicians; and (3) interfaces that are simple, easy to use, and visually intuitive enable use (Textbox 2).

Textbox 2. **Themes and subthemes of Component 2 (digital peer support workshops and interviews).**

**Theme 1–User choice and autonomy are essential to promote engagement**

- Peers want to determine their own level of privacy

*“Look, I think a picture and a name would help but I'’m mindful of some of the issues may be around that….the potential issues around confidentiality. There’s always a risk of inappropriate behavior.*” [Participant 13, male, exercise physiologist]
*“I don’t want a photo of myself, my name would be fine. They don’t know my surname so I'’m anonymous to them anyway. So, in that sense anonymous but I am who I am, I’m not making myself somebody else.*” [Participant 1, female, consumer]

- Pre-establishing hierarchy disengages users

*“I have a real problem with [the terminology] ‘child.’ It needs to be buddy, or mentor, or guardian buddy, or something that’s a bit more, working together.*” [Participant 1, female, consumer]

- Mandatory commenting is not likely to improve engagement

*“The patient should be allowed to decide what they want. Maybe some of them are just happy to go on and [not actively participate] but I think….that’s maybe an option.*” [Participant 7, female, registered nurse]
*“I can’t imagine [being compelled into] communicating, I have no need to write a note to someone to say I feel bad today or I feel good. I just don’t*.” [Participant 2, male, consumer]

- Interventions may benefit users more when there is versatility in using in conjunction with routine care

*“We have a lot of people who return to work around that eight-week mark. They’re even rushing to get through. Sometimes we can finish up a little bit early if they’re returning to work. So certainly, the population, the healthy, younger, returning to full time work population, would find an app quite a flexible way to be able to keep connected.*” [Participant 12, female, physiotherapist]

**Theme 2–Safeguarding is important to both users and clinicians**

- Clear rules and disclaimers are necessary to establish safe use and expectations

*“I want to leave it up to individuals to be in charge themselves and be able to step back, but there’s always that concern. I think if you‘re going to try and organize peer support online, you’ve got to set some ground rules. The Facebook site that we have, we have guidelines as to what’s okay and what’s not. I would imagine Facebook probably has their own [too]….*” [Participant 7, female, registered nurse]
*“I have a question; how do you guard against abuse? [Interviewer: Are you thinking we need a moderator?] Possibly, because it’s open to abuse, isn’t it? [Interviewer: Yes. There’s a few different things including that front conditions that you accept. We can add a bit more of a checklist in there. Would that feel comfortable?] I think probably. Maybe you would have to have a report function, wouldn’t you?*” [Participant 1, female, consumer]

- Moderation by credentialed clinicians is needed to screen for information inaccuracy or harm

*“What about moderating rather than interceding, interfering. There’s pros and cons to that. I mean….there has to be a degree of ‘trust me.’ But….I think it needs to be monitored, because there is the potential for all sorts of crazy stuff. It can be an honest mistake, but it could also be bullying.*” [Participant 11, female, cardiologist]
*“….if a [peer] sent me a question like ‘I forgot to take medication this morning, is it ok to take now?’ then I would not answer. I would say, you should refer to [a health care professional].*” [Participant 4, male, consumer]

**Theme 3 – Interfaces that are simple, easy to use, and visually intuitive enable use**

- Steps that are complicated or not intuitive are a point of disengagement

*“Why hasn’t it got space written in there? Old people like me don’t know that! Also, [it] doesn'’t automatically give you via capitals for your surname; in this day and age should be automatic. Am I a pain? And also emails, most of them don’t have uppercase when in the start of their email, so I find that very aggravating….you got to go back again….to change it to lowercase!*” [Participant 2, male, consumer]
*“….if someone set it up for him on the iPad, he could actually….he would know how to use it*” [Participant 8, female, epidemiologist]

- Interfaces using instinctive icons, concise messaging, and understandable data are easier to use

*“I like that because I think that’s short and sharp. Not too much information, and putting it in it’s quite quick.*” [Participant 1, female, consumer]
*“It would be great if they put in more, but [it’s] tricky….complex….I like it with just fruit and vegetables.*” [Participant 9, female, dietician]

- Group interfaces allowing authentic relationships to form are preferred over one-to-one peer matching

*“….we let them do it themselves, rather than sort of playing matchmaker. I think it works better if there’s a natural evolution of relationship with between patients.*” [Participant 13, male, exercise physiologist]
*“I think some support would be alright but I wouldn’t be necessarily comfortable with just me because we’ve all got such different issues.*” [Participant 5, female, consumer]

- Interfaces that are tailored and pertinent to the user increase engagement

*“It has to be individually tailored, even if it’s all algorithm driven….[ie,] ‘dear [name]’ or whatever, anything that’s individualized or personalized, increases acceptability and engagement for the user.*” [Participant 10, female, registered nurse]
*“[interviewer: what would you log?] Well, we [I] wouldn’t go to smoking, would we! [non-smoker]*” [Participant 2, male, consumer]

#### Theme 1: User Choice and Autonomy Are Essential to Promote Engagement

User choice and autonomy were frequently identified as important considerations in the development of digital peer support interventions. This included the option to allow users to pick a naming convention they were comfortable with (eg, first name only, full name, pseudonym, or username) and the option to display a picture, an avatar, or neither. Aside from user options within the interface of the app, consumers and clinicians identified the advantages in making digital peer support flexible for participation at all stages of recovery, including having the choice to use it alongside routine care and cardiac rehabilitation. Having a choice to not comment or contribute to discussions (ie, passive participation) was also identified as an important autonomous factor, as participants acknowledged that prospective app users could still derive benefit from observing without interacting. Feedback from participants and clinicians strongly expressed a dislike of the use of any hierarchical language (in this case supporter [“guardian angel”] vs beneficiary [“child”]), instead advocating for inclusive nonhierarchical language (ie, “mate,” “buddy,” and “supporter”) that reflected unforced natural relationships, and which also acknowledged all peer experiences as equal and valid.

#### Theme 2: Safeguarding Is Important to Both Users and Clinicians

Consumers and clinicians identified the importance of clear rules and disclaimers for safeguarding, especially specifying the hours that the program will be moderated. Consumers identified rules and moderation as a necessity, with some acknowledging the need to feel protected from harm, bullying, and incorrect information, and others noting that they would feel uncomfortable offering information to peers in an environment that was unmoderated. Experts highlighted observations from their clinical practice that emphasized the potential for problems to occur where rules, disclaimers, and moderation are not used. Both consumers and experts felt that moderation should be undertaken by a credentialed clinician taking a largely passive role, intervening whenever appropriate.

#### Themes 3: Interfaces That Are Simple, Easy to Use, and Visually Intuitive Enable Use

Participants described the need for interfaces to be simple and uncomplicated to encourage use, including easy setup and access, with intuitive visuals and icons, and short, concise messaging to not overwhelm users. Both consumers and experts alike described a need for digital peer support interfaces to be tailored to the user, to maximize engagement and provide a personalized experience. Participants reflected that interfaces featuring progress tracking or comparison between peers should allow data to be entered and presented in a format that is easily understood by users (eg, whole cigarettes instead of units of tobacco). Consumers and clinicians alike identified that interventions designed with a group-based or forum format would be preferable over one-to-one peer “matching” models, as this allows users to form their own authentic relationships in group settings, emphasizing the importance of establishing nonhierarchical relationships, as discussed in Theme 1.

### Framework of Recommendations for Digital Peer Support Development and Delivery

**Figure 1.**
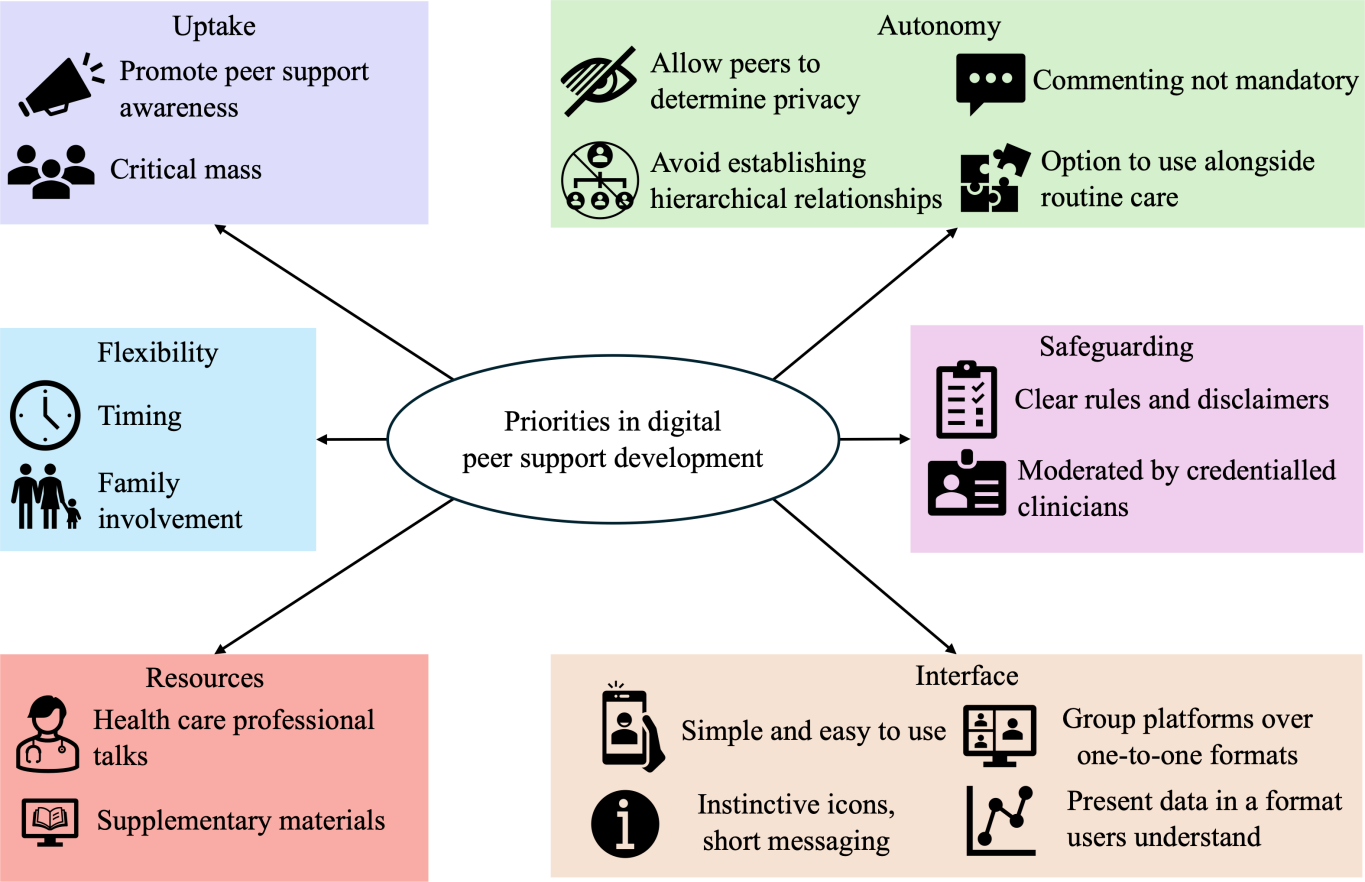
Framework for developers of digital peer support programs.

Synthesis of preferences identified from in-person peer support attenders (Component 1) and feedback from consumer workshops and clinician interviews (Component 2) informed the development of this framework of recommendations for future peer support development, comprised of 6 focused areas: uptake, flexibility, resources, autonomy, safeguarding, and interface (Figure 1). Greater peer support awareness to encourage participation and thus provide sufficient numbers (ie, “critical mass”) is needed for interventions to work effectively. Flexibility in the timing of the intervention and options of family involvement, resources including health care professional input and supplementary materials, and simple, tailorable, visually instinctive platforms that allow for group interaction are all elements that should be considered to increase engagement. Autonomy of the user is a key consideration for developers and should allow users a range of choices, including determining levels of privacy, establishing their own (nonhierarchical) relationships (preferably in a group setting), commenting if and when they choose, and having the option to use the intervention in conjunction with routine care. Safeguarding should be ensured for users of the intervention as well as the clinicians overseeing the moderation (ie, to address liability), which can be addressed by providing clearly stated rules to guide participants, disclaimers of when and how the intervention will be moderated, and the credentials of the clinician providing the moderation.

## Discussion

### Principal Findings

This research identifies the multiple benefits of peer support for people with CVD and provides priorities to consider in the development of future digital peer support interventions. The benefits identified for patients with CVD were grounded in peers sharing experiences and included a way of coping with diagnosis and recovery, a forum for experiential learning, a mutual understanding of living with CVD, and access to a supportive community of similar individuals. Future priorities identified for peer support groups from current attenders included improving awareness to ensure peer support uptake, flexibility with timing and family involvement, and health care professional input for resources and adequate safeguarding. Feedback from consumers and experts using a digital peer support prototype indicated preferences for future digital peer support to include autonomous features and use, safeguarding by credentialed health care professionals, and simple-to-use, personalized interfaces. Overall, digital peer support developers are recommended to ensure that uptake, flexibility, resources, autonomy, safeguarding, and easy-to-use interfaces are prioritized.

Flexibility, autonomy, and customizability were identified as important concepts for digital peer support strategies. Across peer support and multiple digital health contexts, tailored and personalized interventions are crucial to maximize engagement [31-33]. Flexibility in scheduling and delivery has been identified as a crucial component of peer support success [17], promoting access to and uptake of peer support programs [34], and is a priority of peers in the peer support literature [31]. Compared to in-person formats, digital interventions for CVD self-management, including cardiac telerehabilitation, are advantageous because of the flexibility, convenience, and easy access that result from virtual delivery [35]. However, digital delivery is not suited to everyone; for example, some participants in a cardiac telerehabilitation program noted that virtual interaction “isn’t the same” as an in-person connection or were unable to navigate technical issues [36]. Therefore, the flexibility to use interventions in conjunction with other digital, in-person, and hybrid peer support models, and alongside or in place of cardiac rehabilitation, is an important consideration. Flexibility around family and caregiver inclusion and programming has also been identified as a priority in peer support settings [31]. Autonomy in modifying anonymity within digital peer support interventions was a key priority identified and may be contingent on the format of the intervention. For example, while some participants in our study noted that they would prefer maintaining some elements of personal identity, anonymity has been reported as an enabler of engagement in online digital peer support forums [37]. Having the autonomy to decide whether or not to comment or engage with a particular post is also supported by the wider peer support literature, which suggests that peers also derive benefit from passive participation [38], known as “lurking,” which contributes to online information dissemination [39]. Thus, evidence suggests that the number of comments or interactions (eg, “likes”) on any given post is not necessarily an exact reflection of engagement [40], and this is reflected in online digital support group research, where 85% of the sample engaged in passive participation [38].

Participants highlighted the need for greater peer support awareness and the importance of a critical mass of participants. The absence of numbers in groups and forums causes disengagement, referred to as the “empty room” phenomenon, and highlights the importance of a critical mass for benefits [40]. This is consistent with the wider peer support literature in which awareness and reach are considered priorities and draws parallels with established peer support groups, which often have to rely on peer support volunteers as a means of referral and awareness [41]. This is problematic, as barriers to peer volunteers in hospitals exist, including negative staff attitudes and ambiguity around the use of peers and the role they can take [42]. Aside from peer volunteers, even in clinical settings such as oncology, where peer support is generally promoted by clinical staff, directing patients toward peer support services is not regarded as common practice [43]. Suggestions to improve referrals have included peer support awareness campaigns targeting clinicians [34] and social media as a referral and promotion tool [31]. However, a further issue is that clinicians have reported reservations around the accuracy of peer-shared information [41,43]. Although research exploring unmoderated health forums does not necessarily validate these concerns [44], there are no supporting studies demonstrating that unmoderated, unstructured, or internet-based digital peer support groups significantly improve outcomes such as quality of life or emotional distress [10]. Furthermore, although individuals regard information provided by their peers as credible and low risk of harm, it is not always considered as trustworthy as that received from a health care provider [45]. As such, this evidence supports the recommendation of moderation by a credentialed health care professional, as identified in this study, which is also advocated in the broader literature [10,40,46]. Interestingly, moderation was not identified as an issue for in-person peer support attenders, perhaps because peer leaders typically undergo training in facilitation [27]. More research is needed to understand the role of moderation in peer support.

These findings indicate that peer support has several benefits that are valuable in CVD recovery and support, which can be understood within the scope of several established theories. For instance, social cognitive theory identifies that learning occurs in a social environment where people can bidirectionally influence one another [47], reflecting how participants acknowledged that peer support provides a way of coping and learning from each other. Another example is social comparison theory, which postulates that people who share similar characteristics (such as a diagnosis) are more likely to gain optimism, motivation, and be at ease with each other [48]. This theoretical lens is useful for comprehending why participants felt like they understood what other peers were going through and why they reported that the peer community uplifts mood and builds confidence. People with CVD have diverse recovery experiences, which differ during stages of disease [49], and many need support earlier on in managing expectations, regaining control of health, and self-management [33]. Barriers to efficient self-management of CVD include unmet informational needs, psychosocial and mental health impacts [50], and difficulty accessing health care [33]. Even though readiness for information varies between individuals following CVD diagnosis, evidence suggests that many patients process, prefer, or receive less information during inpatient admission [51,52]. Peer support may therefore fill these gaps in ongoing CVD care because coping (especially with psychosocial impacts), learning, understanding, and peer interaction are nurtured. Peer support cultivates a sense of community and shared understanding, which is beneficial in secondary prevention because shared engagement in health activities is an effective self-management motivator [50]. Shared understanding avoided misunderstanding or judgment from family and friends, which can have a detrimental influence [50], while fostering authentic friendships, which contribute to seeing recovery as a shared journey [53]. Frequent “checking in” is important for behavior change sustainment, but continuity of professional support persists as a challenge in ongoing CVD care [50]; therefore, peer support may be a beneficial substitute, with peers keeping each other accountable and sharing encouragement on the recovery journey.

### Limitations

Some limitations of this study should be noted. To the best of our knowledge, this is the first study to explore the views on important elements of peer support in CVD and priorities for digital strategies. However, as a qualitative study, these results are derived from a limited sample and are subjective; therefore, causation cannot be inferred, and future research is required to further explore these findings. The participants in the in-person focus groups (Component 1) were already attending peer support programs and were therefore likely to be positively biased toward the benefits. Further research is required to seek the experiences and opinions of consumers with CVD who do not attend, or have not yet joined, a peer support group. The consumers in Component 2 were a small sample that tested a prototype app in an exploratory, focus group–style run workshop, the purpose of which was to generate feedback, ideas, and priorities. Therefore, these results should be interpreted with caution when considering the generalizability to broader and more diverse populations. This prototype was used for exploratory purposes only and is not commercially available, and consumers in Component 2 did not have prolonged exposure to a peer support environment. Further research is needed to develop and test digital peer support strategies for CVD populations. Finally, practical implications for real-world adoption, such as cost, clinician training, and integration with existing health systems and programs, did not emerge from the clinician interviews. These are important factors for scaling up and implementation of peer support interventions and warrant further investigation.

### Conclusion

People with CVD attending peer support report many benefits that are obtained through shared experiences and that result in coping, learning, feeling understood and validated, and a sense of community. Ensuring peer support awareness and uptake, flexibility with timing and family involvement, and health care professional involvement and provision of resources were identified as important priorities by those already attending peer support programs. Autonomous features that enable choice, adequate safeguarding using checklists and credentialed health care professionals, and easy-to-use interfaces were identified as priorities by consumers and clinicians testing a digital peer support prototype. Collectively, these priorities constitute a framework of important elements that should take precedence when developing digital peer support interventions. These findings demonstrate the importance of consultation with the group concerned when developing peer support interventions, indicating that there should ideally be a co-design process, or at a minimum, consultation in the development of such interventions. When testing newly designed peer support interventions, feedback can be sought in relation to these key areas that were prioritized. Future interventions are then ideally placed to test the extent of the difference these priorities make to consumers engaged in peer support.

## Supplementary material

10.2196/72743 Multimedia Appendix 1 Example quotations for coding of themes and subthemes.

10.2196/72743 Multimedia Appendix 2 Characteristics of participants of Component 2 (n=13).

10.2196/72743 Checklist 1 COREQ checklist.

## Abbreviations

CHD: coronary heart disease
COREQ: Consolidated Criteria for Reporting Qualitative Research
CVD: cardiovascular disease

## References

[1] Lindstrom M, DeCleene N, Dorsey H (2022). Global burden of cardiovascular diseases and risks collaboration, 1990-2021. J Am Coll Cardiol.

[2] de Oliveira L, de Carvalho Costa I, da Silva DG (2019). Readmission of patients with acute coronary syndrome and determinants. Arq Bras Cardiol.

[3] Ambrosetti M, Abreu A, Corrà U (2021). Secondary prevention through comprehensive cardiovascular rehabilitation: From knowledge to implementation. 2020 update. A position paper from the Secondary Prevention and Rehabilitation Section of the European Association of Preventive Cardiology. Eur J Prev Cardiol.

[4] Byrne RA, Rossello X, Coughlan JJ (2023). 2023 ESC guidelines for the management of acute coronary syndromes. Eur Heart J.

[5] Pedersen M, Støier L, Egerod I, Overgaard D (2021). Mastery of everyday life and social support needs in older vulnerable women with myocardial infarction and their relatives: a qualitative study. Eur J Cardiovasc Nurs.

[6] Isaksen AS, Gjengedal E (2006). Significance of fellow patients for patients with myocardial infarction. Scand J Caring Sci.

[7] Benzer W, Rauch B, Schmid JP (2017). Exercise-based cardiac rehabilitation in twelve European countries results of the European Cardiac Rehabilitation Registry. Int J Cardiol.

[8] Xu Z, Liu X, Ghisi G de M (2021). Impacts of the COVID-19 pandemic on cardiac rehabilitation delivery around the world. Glob Heart.

[9] Ferrel-Yui D, Candelaria D, Pettersen TR, Gallagher R, Shi W (2024). Uptake and implementation of cardiac telerehabilitation: a systematic review of provider and system barriers and enablers. Int J Med Inform.

[10] Hu J, Wang X, Guo S (2019). Peer support interventions for breast cancer patients: a systematic review. Breast Cancer Res Treat.

[11] Maguire R, Hanly P, Maguire P (2021). Living well with chronic illness: How social support, loneliness and psychological appraisals relate to well-being in a population-based European sample. J Health Psychol.

[12] Mookadam F, Arthur HM (2004). Social support and its relationship to morbidity and mortality after acute myocardial infarction: systematic overview. Arch Intern Med.

[13] Joekes K, Maes S, Warrens M (2007). Predicting quality of life and self-management from dyadic support and overprotection after myocardial infarction. Br J Health Psychol.

[14] Harris GE, Larsen D (2007). HIV peer counseling and the development of hope: perspectives from peer counselors and peer counseling recipients. AIDS Patient Care STDS.

[15] Embuldeniya G, Veinot P, Bell E (2013). The experience and impact of chronic disease peer support interventions: a qualitative synthesis. Patient Educ Couns.

[16] Karp N, Yazdany J, Schmajuk G (2023). Peer support in rheumatic diseases: a narrative literature review. Patient Prefer Adherence.

[17] Wasilewski MB, Rios J, Simpson R (2023). Peer support for traumatic injury survivors: a scoping review. Disabil Rehabil.

[18] Chen C, Zhou Y, Xu JY, Song HY, Yin XW, Gu ZJ (2024). Effect of peer support interventions in patients with type 2 diabetes: a systematic review. Patient Educ Couns.

[19] Zhang S, Li J, Hu X (2022). Peer support interventions on quality of life, depression, anxiety, and self-efficacy among patients with cancer: a systematic review and meta-analysis. Patient Educ Couns.

[20] Marshall P, Booth M, Coole M (2024). Understanding the impacts of online mental health peer support forums: realist synthesis. JMIR Ment Health.

[21] Olsson M, Eliasson I, Kautsky S, Hård Af Segerstad Y, Nilsson S (2024). Co-creation of a digital platform for peer support in a community of adolescent and young adult patients during and after cancer. Eur J Oncol Nurs.

[22] Kong LN, Hu P, Yang L, Cui D (2019). The effectiveness of peer support on self-efficacy and quality of life in adults with type 2 diabetes: a systematic review and meta-analysis. J Adv Nurs.

[23] Hildingh C, Fridlund B (2004). A 3-year follow-up of participation in peer support groups after a cardiac event. Eur J Cardiovasc Nurs.

[24] Weddell J, Shi W, Redfern J, Buckley T, Gallagher R (2025). Effectiveness of coronary heart disease peer support interventions: a systematic review and meta-analysis. Eur J Prev Cardiol.

[25] Rayland A, Andrews J (2023). From social network to peer support network: opportunities to explore mechanisms of online peer support for mental health. JMIR Ment Health.

[26] Evans M, Cuddeback GS, Golin C (2025). Diverse elements comprising studies of peer support complicate evidence synthesis. J Ment Health.

[27] Home. Heart Support Australia.

[28] Braun V, Clarke V (2022). Thematic Analysis: A Practical Guide.

[29] Saunders B, Sim J, Kingstone T (2018). Saturation in qualitative research: exploring its conceptualization and operationalization. Qual Quant.

[30] Tong A, Sainsbury P, Craig J (2007). Consolidated criteria for reporting qualitative research (COREQ): a 32-item checklist for interviews and focus groups. Int J Qual Health Care.

[31] Elliott MJ, Donald M, Farragher J (2023). Priorities for peer support delivery among adults living with chronic kidney disease: a patient-oriented consensus workshop. CMAJ Open.

[32] Redfern J, Santo K, Coorey G (2016). Factors influencing engagement, perceived usefulness and behavioral mechanisms associated with a text message support program. PLoS ONE.

[33] Vosbergen S, Janzen J, Stappers PJ (2013). A qualitative participatory study to identify experiences of coronary heart disease patients to support the development of online self-management services. Int J Med Inform.

[34] Trasolini A, Wood E, Thomas N (2021). A narrative review of peer support barriers and facilitators in kidney care. J Ren Care.

[35] Subedi N, Rawstorn JC, Gao L, Koorts H, Maddison R (2020). Implementation of telerehabilitation interventions for the self-management of cardiovascular disease: systematic review. JMIR Mhealth Uhealth.

[36] Fuda MR, Patel P, Van Es J (2024). “Comfort of sitting at home while getting information i needed”: experiences of cardiac patients attending virtual cardiac rehabilitation. CJC Open.

[37] Evett D, Hutchinson K, Bierbaum M (2021). Peer support and social network groups among people living with epilepsy: a scoping review. Epilepsy Behav.

[38] Wu N, Wang SJ, Brazeau AS (2023). Supporting and incentivizing peer leaders for an internet-based private peer community for youths with type 1 diabetes: social network and directed content analysis. J Med Internet Res.

[39] Khoshnaw S, Panzarasa P, De Simoni A (2024). Metaphor diffusion in online health communities: infodemiology study in a stroke online health community. JMIR Cardio.

[40] Partridge SR, Gallagher P, Freeman B, Gallagher R (2018). Facebook groups for the management of chronic diseases. J Med Internet Res.

[41] Brodar KE, Carlisle V, Tang PY, Fisher EB (2022). Identification and characterization of peer support for cancer prevention and care: a practice review. J Cancer Educ.

[42] Cooper RE, Saunders KRK, Greenburgh A (2024). The effectiveness, implementation, and experiences of peer support approaches for mental health: a systematic umbrella review. BMC Med.

[43] Kallio R, Jones M, Pietilä I, Harju E (2021). Perspectives of oncology nurses on peer support for patients with cancer. Eur J Oncol Nurs.

[44] Hoffman-Goetz L, Donelle L, Thomson MD (2009). Clinical guidelines about diabetes and the accuracy of peer information in an unmoderated online health forum for retired persons. Inform Health Soc Care.

[45] Litchman ML, Edelman LS (2019). Perceptions of the diabetes online community’s credibility, social capital, and help and harm: cross-sectional comparison between baby boomers and younger adults. JMIR Aging.

[46] Clougher D, Ciria-Suarez L, Medina JC, Anastasiadou D, Racioppi A, Ochoa-Arnedo C (2024). What works in peer support for breast cancer survivors: a qualitative systematic review and meta-ethnography. Appl Psychol Health Well Being.

[47] Bandura A (1986). Social Foundations of Thought and Action: A Social Cognitive Theory.

[48] Festinger L (1954). A theory of social comparison processes. Human Relations.

[49] Decker C, Garavalia L, Chen C (2007). Acute myocardial infarction patients’ information needs over the course of treatment and recovery. J Cardiovasc Nurs.

[50] Cohen Rodrigues TR, de Buisonjé DR, Keesman M (2021). Facilitators of and barriers to lifestyle support and eHealth solutions: interview study among health care professionals working in cardiac care. J Med Internet Res.

[51] Zwack CC, Smith C, Poulsen V, Raffoul N, Redfern J (2023). Information needs and communication strategies for people with coronary heart disease: a scoping review. Int J Environ Res Public Health.

[52] Weddell J, Naismith SL, Bauman A (2023). Age and marital status predict mild cognitive impairment during acute coronary syndrome admission: an observational study of acute coronary syndrome inpatients. J Cardiovasc Nurs.

[53] May C, Bieber K, Chow D, Mortenson WB, Schmidt J (2023). Experiences of adults with stroke attending a peer-led peer-support group. Brain Impair.

