# Supplementary material for: Framework for the Development and Delivery of Digital Peer Support Programs: Qualitative Study on in-Person and Digital Delivery for People With Cardiovascular Disease
Source: J Med Internet Res. 2025 Oct 16;27:e72743. doi: 10.2196/72743 (PMC12530454; doi:10.2196/72743)
Supplement: Multimedia Appendix 1 [file jmir-v27-e72743-s001.docx]

| **Component one** | |
| --- | --- |
| 1 | Peer support provides a way of coping |
| 1.1 | Helps to deal with psychological impacts |
|  | Participant 15, male's gone through the same thing as obviously I have. He said he had the same experience and just talking to him and him telling me why his kidneys were failing or playing up actually lifted me. So it does, this is huge for mental health, absolutely massive for me, from my perspective. – Participant 17, male |
|  | People can actually say exactly what they think. And it's not so much the physical. It's all about getting your head sorted, getting the emotion sorted, and everybody knowing where they're headed. – Participant 15, male |
|  | He said I felt really awful, I kept telling my wife I was depressed. I said, well, being depressed afterwards or feeling low is absolutely normal. And he said, oh, really? No one had actually told him before and he didn't have the benefit of a group like this. – Participant 2, male |
| 1.2 | Talking about experiences provides “cathartic relief” |
|  | …looked at from a peer support, it means you've got a group of people who can get together, basically share their experience, and just let it out. And I think that forms a really unique sort of bond. – Participant 15, male |
|  | To share those experiences I think is a cathartic one. – Participant 9, male |
| 1.3 | Hearing from peers help normalise individuals’ own experiences |
|  | Nobody had bothered to talk to me about the fact that yes, it was going to hurt being split open and how to handle that, and nobody else spoke about the mental side of things, and the emotional impact this could have. – Participant 15, male |
|  | …not everybody would understand, because they haven't had that experience. So at least everybody here, you know, in one shape or form has experienced that similar thing. – Participant 5, male |
| 2 | “We all learn from each other” |
| 2.1 | You find out from peers what “nobody bothered telling me” |
|  | The reason I joined [peers support] is because there were things I wish somebody had bothered to tell me, that I hadn't been told. So, I thought, I need to start telling people. – Participant 15, male |
|  | …they took about two and a half feet of vein out of my left leg. And they didn't say anything much about that at all. Some weeks afterwards, I was having an issue, not with the heart, but where they took the graft out, it was numb and tingling. – Participant 2, male |
| 2.2 | Peer support is useful for helping make sense of hospital admission |
|  | I had my first inkling at about half past eight in the morning, I was in surgery by half past 12 and back home the following day. My biggest problem was saying, what the hell happened to happen so quickly? There was no time for people to impart information, or for me to take that information in and synthesise it, and come up with the logical reasoning. – Participant 9, male |
|  | … people spend a lot less time in hospital. They don’t have enough time to get their head around this. And that’s where the people who have stents and go home quickly are suffering worse. – Participant 15, male |
| 2.3 | Peer support fills shortcomings in patient education |
|  | there is a massive hole in the medical profession for that education, firsthand knowledge, whatever. This fills it. But that medical side, they can't…I don't think they've got the resources. I don't think they've got the time. – Participant 17, male |
|  | [the surgeon] told me after he finished “right, you're good for another 30 years.” So that was encouraging. – Participant 2, male |
|  | The doctors really didn't have a clue. It hadn't happened to them. If it happens to them, they know what to talk about. But when it doesn't happen to them, they've got no idea what [we] go through. – Participant 8, female |
|  | They say don't be Dr. Google and look for yourselves. But where else do you go? You know, go to professional and you don't always feel that they're giving you answers which make sense. – Participant 21, female |
| 3 | My peers understand what I am going through |
| 3.1 | We don’t have to worry family unnecessarily |
|  | There are obviously things that it's better if you [unload] to somebody totally outside of the family. This is where I think it's important to have the contact. You can just pick up the phone and go, I need to talk to someone, and you're not bothering… you're not worrying your family unnecessarily. You can talk to somebody who's been through the same thing but your family isn't getting all concerned. And I think that's important. – Participant 15, male |
|  | We'd had nothing like this in our family, and as I said to be able to accept the fact that I there was people out there that…[Interviewer: That understood you?] Yes, not cared - understood. Because you got all the care in the world from your family, but no one can actually tell you what [you] feel. – Participant 6, female |
|  | Family, they don't understand sometimes, so you've got to explain to them because they're not involved in [your] experience. [So] this is very important, the peer to peer conversation. – Participant 10, male |
|  | It's the fact that the group are people who have been through a similar experience, no matter how caring your family are, they didn't go through the experience with you. Even my wife will say that she doesn't really understand what I went through. Whereas I can go along to the Peer Support group and I know that everybody in that group understands, you don't have to explain to anybody. – Participant 9, male |
| 3.2 | Experience and advice is valued because it comes from a peer |
|  | Society in general does that [judges]. After my heart attack, [people were] saying, oh, that's because you're unhealthy and whatever, and I wasn't, I was fit as a fiddle. But I think genetics is a big thing. Genetics with, I think stress is massive on this stuff. That's what I put mine down to. – Participant 17, male |
|  | When I had my bypass, everyone said how could you? A fruit and veggie eater…but my father and now my daughter has already had her heart attacks start. So [for me] it is definitely a genetic problem. – Participant 8, female |
|  | I felt like there was a lot of blame, because I was so young…I've given up the smoking. I've given up the drinking. I've already lost 50 kilos. I've done the right things. And then it happened. So I have, and still have, a lot of mental issues and depression about why. – Participant 6, female |
| 3.3 | Peer support is a safe space for asking questions |
|  | We all come to an understanding fairly quickly: there are no stupid questions. If you've got a question, it's obviously worrying you, so it can't be stupid. I think we all understand that. So the questions get asked and some can be quite personal, and that doesn't Participant 17, maleer. – Participant 9, male |
|  | …somebody who's been through it who you can simply ask the question. They might be silly questions. Well, they're questions you won't ask a professional. But you can ask somebody who's been there. – Participant 15, male |
|  | You see reticence initially with new people coming in, [which] you would expect. But what is lovely is to see the way that reticence drops over a couple of months as people become comfortable sharing information with like-minded people, knowing that it's within the confines of the meeting, and that they are understood and appreciated for that insight. – Participant 9, male |
| 4 | Peer support is a community that uplifts mood and builds confidence |
| 4.1 | “People pick you up” |
|  | ‘That's how I feel about [the peer support] group. Sometimes you don’t say anything...you don't need to ask questions or say how your feelings are, because by the end of the group session, you've gotten some information or you feel a little bit light-hearted, and lifted by being around…[Participant: or somebody asks you…] That's right! ‘Are you okay?’’ – Participant 6, female |
| 4.2 | Experiences provide confidence, hope and vicarious self-efficacy |
|  | Sometimes you don't even have to ask. You just listen to other people's stories and what we're talking and it's like, oh! Okay, that makes you feel a bit better…[I might] try that when I get home. I pick up from things like that. – Participant 6, female |
|  | The good thing about it is there's people more advanced…they've had heart attacks long time ago, so we can learn off them just what's in store for us. And seeing somebody, excuse me saying so, old. You're not worried about your life so much. It's not a death sentence. You're on for a while! – Participant 7, male |
|  | It's also a source of reassurance for those of us who are either facing it or have been through it, particularly from people whose event was 25 years ago, still going strong. That's good news for the rest of us. – Participant 2, male |
|  | meeting somebody who's just had the operation was the best thing that could have happened because there was proof [that you can get through it]...Participant 11, male |
| 4.3 | The group helps support each other |
|  | One of the guys he actually rang and said, "Look, my doctors told me I have to stay home” so I just rang to check on him. He lives by himself. So I just rang to make sure he was okay. It's just a Participant 17, maleer of touching base every now and again to make sure people are still functioning. – Participant 15, male |
|  | It becomes like a family. And they're annoying, but they're good [laughter]. – Participant 17, male |
| 5 | Awareness, flexibility and resources are important for engagement |
| 5.1 | Greater peer support awareness is required for uptake |
|  | I would say the hospital's are shit at that. I'll be honest. The only way I found out was from Participant 15, male, who came and did a lecture...The hospital itself gave me a piece of paper with about eight or 80 different names, different organizations like your walking organization. But the hospital never ever mentioned this, which is really a pity. Cause I think everyone in the hospital would benefit from it. – Participant 17, male |
|  | Well I rang the hospital and they didn't know, what it was about. – Participant 1, male |
|  | I wasn't approached in [hospital]. When I was in hospital, I was there for about 10 days. And no one came and told me about the group. So, I must have fallen through the cracks. – Participant 11, male |
|  | I actually Googled to find this group…I just had a defibrillator but I had a frozen shoulder after my six weeks of not being able to lift my arms and all that. I found it really difficult. I kept going back to my doctor and she said she'd ring the rehab, and they said, no, we don't take people who just have defibrillators. I was like, well, what do I do? – Participant 14, female |
|  | We had a way, way traumatic time after the operation and we felt we would have benefited by joining this group, but unfortunately we didn't learn about this until only recently. – Participant 2, male |
|  | And finally, it won't work if you haven't got many people turn up to peer group meetings, because it's the mass of people that make it work. That’s a thank you to all of you who come along here and support people like Alex because you're the guys that make this work. – Participant 9, male |
| 5.2 | Flexibility with timing and family involvement are desirable to maximise reach |
|  | I would like it very early rather than later because we do go through a really bad time, not knowing…understanding what’s happening. And you become very sensitive [to] changes and things that are happening to you. You worry unnecessarily. So you can come in early, that’d be good. – Participant 3, male |
|  | I [didn’t] have the time to have [a hospital peer volunteer] come see me. So after care would be better, but if you could get in contact with someone, rather than just in two weeks time, you can go to this group. – Participant 6, female |
|  | [some people with CVD] still have to work. They still have to mind their children at home or their grandchildren. They haven't got that one day a month that they can go okay, I can definitely do it on the Tuesday. I could definitely do it on Wednesday. – Participant 6, female |
|  | More family members. So that they can learn, so that they can see that their family member is not a special case. That's sort of what I got from it, I come with dad and now I can sort of see that it's a lot more common. – Participant 5, male |
| 5.3 | Health professional involvement provides a talking point |
|  | Yeah we've had doctors come and talk about medication or you know, have set up life plans and all sorts of different things that, you know, we all call us are on medication. So having that information given to us helps. Cause you know, you, are in hospital, they give you a bag of medication and say, Here you go. So you can kill yourself on this. - Participant 17, male |
| 5.4 | Supplementary materials enhances discussions and experience sharing |
|  | Some of these words don’t make sense…It would be useful if you had stuff included. If you are going to use language like, a serve of vegetables, well – what is a serve of vegetables? Links to where some of this stuff is explained at greater length. If that was within the app, a few links to health information. – Participant 4, male |
|  | When we've got the information on pamphlet and we sit around talking about it, it gives you more support for each other. Handouts are always good. You can take them home and read it later again or refer to what someone in your group had said, I'll read about that. – Participant 6, female |
| **Component two** | |
| 1 | Autonomy is essential to promote engagement |
| 1.1 | Peers want to determine their own level of privacy |
|  | Look, I think a picture and a name would help but I'm mindful of some of the issues may be around that…the potential issues around confidentiality. There's always a risk of inappropriate behaviour. – Participant 13, male, exercise physiologist |
|  | I don’t want a photo of myself, my name would be fine. They don’t know my surname so I'm anonymous to them anyway. So, in that sense anonymous but I am who I am, I’m not making myself somebody else – Participant 1, female, consumer |
| 1.2 | Pre-establishing hierarchy disengages users |
|  | I have a real problem with [the terminology] “child”. It needs to be buddy, or mentor, or guardian buddy, or something that’s a bit more, working together. – Participant 1, female, consumer |
|  | I quite like “mate” and I…Ah, [many] ladies use “mate” these days. Well, that’s what you’re trying to determine, isn’t it; a mate, friend, buddy. – Participant 2, male, consumer |
| 1.3 | Mandatory commenting is not likely to improve engagement |
|  | Some people [need contact] 2 weekly, and some people say, I've got all I need. I'll call you…They're kind of essentially signing off, you know? Yeah which is fine. – Participant 7, female, registered nurse |
|  | The patient should be allowed to decide what they want. Maybe some of them are just happy to go on and [not actively participate] but I think…that's maybe an option. – Participant 7, female, registered nurse |
|  | I can’t imagine [being compelled into] communicating, I have no need to write a note to someone to say I feel bad today or I feel good. I just don’t. – Participant 2, male, consumer |
|  | I wouldn't know what to say to someone that was smoking five cigarettes a day. I could probably be extremely judgmental, which wouldn't do any good. – Participant 4, male |
| 1.4 | Interventions may benefit users more when there is versatility in using in conjunction with routine care |
|  | I think it would have to be used in conjunction with other initiatives, obviously cardiac rehab which is the main one, and then potentially out there programme digital support programmes, just because I think the evidence is that apps are good in the beginning but they're not frequently…you know they use heavily for one month and then they people drop off. - Participant 6, female, public health researcher |
|  | We have a lot of people who return to work around that eight week Participant 4, male. So they're even rushing to get through. Sometimes we can finish up a little bit early if they're returning to work. So certainly the population, the healthy, younger, returning to full time work population, would find an app quite a flexible way to be able to keep connected. – Participant 12, female, physiotherapist |
|  | I think an app is a great it's a bit like telehealth, it's a great sort of adjunct to, or it's another option that people can choose. And some people love that. – Participant 7, female, registered nurse |
|  | I think that to talk to other people is really important. That's an important part of face to face cardiac rehab, so I'm not sure whether having an app on top…would be of extra use at that point in time. But certainly when they go into the community, I think that would be a really good time for them to stay connected, because that's when those bonds really fall off if they're not attending a maintenance programme after they finish. – Participant 12, female, physiotherapist |
| 2 | Safeguarding is important to both users and clinicians |
| 2.1 | Clear rules and disclaimers are necessary to establish safe use and expectations |
|  | I want to leave it up to individuals to be in charge themselves and be able to step back, but there's always that concern. So I think if you're going to try and organise peer support online, you've got to set some ground rules. The Facebook site that we have, we have guidelines as to what's okay and what's not. Okay, and I would imagine Facebook probably has their own, but just sort of talk to people. So this is, yeah, this is an option, but I do think you'd probably have to have some level of supervision at least initially. – Participant 7, female, registered nurse |
|  | Participant 1, female, consumer: I have a question. How do you guard against abuse?  Interviewer: Are you thinking we need a moderator?  Participant 1, female, consumer: Possibly, because it's open to abuse, isn't it?  Interviewer: Yes. There's a few different things including that front conditions that you accept. [We] can add a bit more of a checklist in there. Would that feel comfortable for you, if you had a bit of a checklist?  Participant 1, female, consumer: I think probably, maybe you would have to have a report function, wouldn't you? |
| 2.2 | Moderation by credentialled clinicians is needed to screen for information inaccuracy or harm |
|  | I think someone [health care professional] should be able to scan and read them to make sure that they're appropriate once a week, or whether the app people can help you set up some sort of trigger words or something like that. When you can go through and read if they are giving inappropriate medical advice. – Participant 6, female, public health researcher |
|  | if there's an inappropriate posts, or somebody who's obviously really anxious and needs a bit of support, then I'd rather get that post removed and then speak to them individually, rather than freaking the whole group. – Participant 7, female, registered nurse |
|  | What about moderating rather than interceding, interfering. There's pros and cons to that. I mean…there has to be a degree of ‘trust me’. But…I think it needs to be monitored, because there is the potential for all sorts of crazy stuff. It can be an honest mistake, but it could also be bullying. – Participant 11, female, cardiologist |
|  | …if a child sent me a question like “I forgot to take medication this morning, is it ok to take now?” then I would not answer. I would say, you should refer to [a healthcare professional]. – Participant 4, male |
|  | …it’s a bit weird having an emotional response to not taking medicine. I have forgotten occasionally, which is just a feeling of mild embarrassment. The obvious question is, is it alright if I take this particular pill in the afternoon, since I've forgotten to take it in the morning which is something you want to ask an expert rather than a peer. – Participant 4, male |
| 3 | Interfaces that are simple, easy to use and visually instinctive enable use |
| 3.1 | Steps that are complicated or not intuitive are a point of disengagement |
|  | That's me fiddling. [Interviewer: that's what we all do, you know?] It’s how we learn! - Participant 2, male, consumer |
|  | I’m talking about [the] keyboard here. I'm not sure how easy it is to do, but it’s asking me for my email, and sometimes when entering email stuff, it's got all the icons for the email on the front screen, and that is so handy as opposed to having to switch between…the @ symbols, I’ve got an underscore in my address… - Participant 1, female, consumer |
|  | …because sometimes things get to is too much information is required. They don't do it. - Participant 7, female, registered nurse |
|  | Why hasn’t it got space written in there, old people like me don’t know that. Also doesn't automatically give you via capitals for your surname, in this day and age should be automatic. Am I a pain? And also emails, most of them don't have uppercase when in the start of their email, so I find that very aggravating because 99% are lower case. And then, you gotta go back again, look at that arrow to change it to lowercase! – Participant 2, male, consumer |
|  | Interviewer: So (participant 1) is looking at the opening screen. Is Participant 2, male, consumer looking at the screen now? Yeah. The first screen which gave the instruction. Did you just flick through it?  Participant 1, female, consumer: I just flicked through it and pressed…  Interviewer: So you just ignored it and pressed understand. So that's how people use it…  Participant 1, female, consumer: That's how people use it, yeah. I probably should’ve read it, shouldn’t I?  Interviewer: Well no, no…so, just to be clear, most people are just going to go accept.  All participants: Yeah [unanimous] |
| 3.2 | Interfaces using instinctive icons, concise messaging and understandable data are easier to use |
|  | I like that because I think that's short and sharp. I think that's not too much information, and putting it in, it’s quite quick - Participant 1, female, consumer |
|  | I do think that patients get information overload – Participant 7, female, registered nurse |
|  | Interviewer: So can you click on the icon ‘medications’. Do you think that looks like medications?  Participant 2, male, consumer: No.  Interviewer: So what do you think that looks like?  Participant 2, male, consumer: A lock and a circle. |
| 3.3 | Group interfaces allowing authentic relationships to form are preferred over one-to-one peer matching |
|  | Yeah, clinically and we let them do it themselves, rather than sort of playing matchmaker. I think it works better if there's a natural evolution of relationship with between patients. – Participant 13, male, exercise physiologist |
|  | I do that from time to time when I've got a patient with defib ICD support group. If they want to speak to somebody, then I try and find somebody similar age, similar diagnosis and…what sort of person they are. But you are always mindful that you don't want to …sort of make the support person…give them a lot of responsibility or put too much on them. So it's not easy. What I am trying to do in the education is get them to talk amongst themselves. It just gives them that opportunity. – Participant 7, female, registered nurse |
|  | I think some support would be alright but I wouldn’t be necessarily comfortable with just me because we've all got such different issues. – Participant 5, female, consumer |
| 3.4 | Interfaces that are tailored and pertinent to the user increase engagement |
|  | So if we come to the tenth, which is today, I may have this morning walked 10 kilometres. So I click on the ten…because I don't go by distance so much as time, because the more I make my legs pump, or walk, the more blood I have to pump, that's my theory…so time is my measure of exercise…So that's today. – Participant 2, male, consumer |
