# Supplementary material for: Framework for the Development and Delivery of Digital Peer Support Programs: Qualitative Study on in-Person and Digital Delivery for People With Cardiovascular Disease
Source: J Med Internet Res. 2025 Oct 16;27:e72743. doi: 10.2196/72743 (PMC12530454; doi:10.2196/72743)
Supplement: Multimedia Appendix 2 [file jmir-v27-e72743-s002.docx]

| **Consumers n=5** | | **n** | **%** |
| --- | --- | --- | --- |
| *Age range (years)* | |  | |
|  | 55-64 | 2 | 40 |
|  | 65-74 | 3 | 60 |
| *Male* | | 3 | 60 |
| *Education* | |  | |
|  | ≤ Year 12 | 1 | 20 |
|  | Undergraduate | 3 | 60 |
|  | Postgraduate | 1 | 20 |
| *Marital status* | |  | |
|  | Married/partnered | 4 | 80 |
|  | Single | 1 | 20 |
| *Employment* | |  | |
|  | Retired | 4 | 80 |
|  | Employed | 1 | 20 |
| **Clinicians/researchers n=8** | |  | |
| *Age range (years)* | |  |  |
|  | *30-34* | 2 | 25 |
|  | *35-44* | 1 | 13 |
|  | *45-54* | 2 | 25 |
|  | *55-65* | 3 | 38 |
| *Female* | | 7 | 90 |
| *Mean experience (years)* | | 11.6 | SD 5.9 |
| *Type of expertise* | |  | |
|  | Registered nurse | 2 | 25 |
|  | Cardiologist | 1 | 12 |
|  | Dietician | 1 | 12 |
|  | Epidemiologist | 1 | 12 |
|  | Exercise physiologist | 1 | 12 |
|  | Physiotherapist | 1 | 12 |
|  | Public health researcher | 1 | 12 |
